# Supplementary material for: Destination choice game: A spatial interaction theory on human mobility
Source: Sci Rep. 2019 Jul 1;9:9466. doi: 10.1038/s41598-019-46026-w (PMC6603030; doi:10.1038/s41598-019-46026-w)
Supplement: Supplementary file 1 — Supplementary Information for Destination Choice Game: A Spatial Interaction Theory on Human Mobility [file 41598_2019_46026_MOESM1_ESM.pdf]

Supplementary Information for Destination Choice Game: A Spatial Interaction Theory on Human Mobility

Xiao-Yong Yan, Tao Zhou

Contents

S1 Additional validation of the DCG model **2**

S2 Derivation of the gravity model using potential game theory **2**

S3 Other derivations of the gravity model **6**

S3.1 Maximum entropy approach 6

S3.2 Deterministic utility theory 7

S3.3 Random utility theory 8

References **9**

S1 Additional validation of the DCG model

We use two types of data, namely, intercity travels and intracity trips, to validate the DCG model. Description of these data sets is given below:

(1) Intercity travels. The data for intercity travels in Japan, U. K. and Belgium are extracted from the Gowalla check-in data set [S1] (https://snap.stanford.edu/data/loc-gowalla.html). Gowalla is a location-based social networking website on which users share their locations when checking in. The data set includes 6,442,890 check-ins of users over the period Feb. 2009 - Oct. 2010. For this data set, we define a user's travel as two consecutive check-ins in different cities.

(2) Intracity trips. The records of intracity trips in New York and Los Angeles are extracted from the Foursquare check-in data set [S2], which contains 73,171 users. We define a user's trip as two consecutive check-ins at different locations (here, the locations are defined as the 2010 census blocks; see https://www.census.gov/geo/maps-data/maps/block/2010/). The total number of trips is 182,033. The data for intracity trips in Oslo, Norway is extracted from the Gowalla check-in data set [S1]. Because of the absence of census blocks and traffic analysis zones in Oslo, we simply partition the city into 88 equal-area square zones, each of which is about 1 km $\times$ 1 km. Each zone is one location in the city.

The estimated model parameters for these data sets are shown in table S1, and the prediction results are shown in Figs S1-S4. Analogous to the results shown in the main text, DCG well predicts the real fluxes, with higher accuracy than other benchmarks subject to the SSI.

We further compare the predictions of the DCG model with other benchmark models in terms of travel distance distribution $P(d)$ and destination attraction distribution $P(D)$. The prediction results are shown in Figs S5-S6. In order to quantitatively compare the prediction accuracy of different models, we perform the two-sample Kolmogorov-Smirnov (KS) test [S3] on the model predicted and observed $P(d)$ and $P(D)$. The results are shown in tables S2-S3, from which we can see that the KS statistics of the DCG model are generally smaller than or closer to that of the gravity model, meaning that the DCG model has relatively high prediction accuracy.

S2 Derivation of the gravity model using potential game theory

Potential game theory originated from the congestion game presented by Rosenthal [S4]. Monderer and Shapley defined exact potential games [S5] in which information concerning

the Nash equilibrium can be incorporated in a potential function. They showed that every exact potential game is isomorphic to a congestion game. In the congestion game model, each player chooses a subset of resources. The benefit associated with each resource is a function of the number of players choosing it. The payoff to a player is the sum of the benefits associated with each resource in his strategy choice. A Nash equilibrium is a selection of strategies for all players such that no players can increase their payoffs by changing their strategies individually. Strategy profiles maximizing the potential function are the Nash equilibria [S6].

From the introduction of the congestion game we can see that the degenerated destination choice game (DDCG) neglecting the crowding effect in the destination is a typical congestion game. Below we will give the process for finding the Nash equilibrium solution of the DDCG by maximizing the potential function of the congestion game.

A congestion game is a tuple $(N, R, (\Psi_k)_{k\in N}, (w_j)_{j\in R})$ [S7], where $N = \{1, \ldots, n\}$ is a set of players (for DDCG, it is the set of O_i travellers starting from origin i), $R = \{1, \ldots, m\}$ is a set of resources (for DDCG, it is the set of destinations), $\Psi_k \subseteq 2^R$ is the strategy space of player k (for DDCG, each player can only choose one destination in a strategy), and w_j is a benefit function associated with resource j (for DDCG, it is the utility function, say $w_j = U_{ij}$). Notice that benefit functions can achieve negative values, representing costs of using resources [S6]. $S = S_1, \ldots, S_n$ is a state of the game in which player k chooses strategy $S_k \in \Psi_k$. For a state S, the *congestion* $n_j(S)$ on resource j is the number of players choosing j. For DDCG the number of travellers choosing destination j is $T_{ij}(S)$. The congestion game is an exact potential game [S5], in which the potential function is defined as

$$\phi(S) = \sum_{j\in R} \sum_{k=1}^{n_j(S)} w_j(k). \tag{S1}$$

For the DDCG with utility function $U_{ij} = \alpha \ln A_j - \beta \ln d_{ij} - \ln T_{ij}$, the potential function is

$$\phi(S) = \sum_{j\in R} \sum_{k=1}^{T_{ij}(S)} U_{ij}(k) = \sum_{j\in R} \sum_{k=1}^{T_{ij}(S)} (\alpha \ln A_j - \beta \ln d_{ij} - \ln k), \tag{S2}$$

where A_j is the attractiveness of location j, d_{ij} is the geometric distance between i and j, and α and β are nonnegative parameters. To find the Nash equilibrium solution of DDCG, we treat T_{ij} as a continuous variable. Then, Eq. (S2) can be rewritten as

$$\phi(S) = \sum_{j} \int_0^{T_{ij}(S)} (\alpha \ln A_j - \beta \ln d_{ij} - \ln x)\mathrm{d}x. \tag{S3}$$

For the optimization problem in which $\max \phi(S)$ is subjected to $\sum_j T_{ij} = O_i$, we can use

the Lagrange multiplier method to obtain the solution. The Lagrangian expression is

$$\max L(T_{ij},\lambda)=\sum_j\int_0^{T_{ij}}(\alpha\ln A_j-\beta\ln d_{ij}-\ln x)\mathrm{d}x+\lambda(\sum_j T_{ij}-O_i), \tag{S4}$$

where λ is a Lagrange multiplier. The partial derivative of the Lagrangian expression with respect to T_{ij} is

$$\frac{\partial L}{\partial T_{ij}}=\alpha\ln A_j-\beta\ln d_{ij}-\ln T_{ij}+\lambda=0, \tag{S5}$$

therefore

$$T_{ij}=\mathrm{e}^{\lambda}A_j^{\alpha}d_{ij}^{-\beta}. \tag{S6}$$

Another partial derivative is

$$\frac{\partial L}{\partial \lambda}=\sum_j T_{ij}-O_i=0. \tag{S7}$$

From Eq. (S6) and Eq. (S7) we can get

$$\sum_j \mathrm{e}^{\lambda}A_j^{\alpha}d_{ij}^{-\beta}-O_i=0, \tag{S8}$$

or

$$\mathrm{e}^{\lambda}=\frac{O_i}{\sum_j A_j^{\alpha}d_{ij}^{-\beta}} \tag{S9}$$

By combining Eq. (S9) and Eq. (S6) we can derive

$$T_{ij}=O_i\frac{A_j^{\alpha}d_{ij}^{-\beta}}{\sum_j A_j^{\alpha}d_{ij}^{-\beta}}, \tag{S10}$$

which happens to be an origin-constrained gravity model with two free parameters. If we set $\alpha=1$, the solution becomes

$$T_{ij}=O_i\frac{A_j d_{ij}^{-\beta}}{\sum_j A_j d_{ij}^{-\beta}}, \tag{S11}$$

which is the standard origin-constrained gravity model [S8].

Now back to the DCG model that considers both the congestion on the way and the crowding in the destination. Its utility function is $U_{ij}=\alpha\ln A_j-\beta\ln d_{ij}-\gamma\ln D_j-\ln T_{ij}$. If the crowding cost $\ln D_j$ is not affected by the fluxes T_{ij}, the maximization of the potential function $\phi(S)=\sum_j\int_0^{T_{ij}}(\alpha\ln A_j-\beta\ln d_{ij}-\gamma\ln D_j-\ln x)\mathrm{d}x$ leads to the following result

$$T_{ij}=O_i\frac{A_j^{\alpha}d_{ij}^{-\beta}D_j^{-\gamma}}{\sum_j A_j^{\alpha}d_{ij}^{-\beta}D_j^{-\gamma}}. \tag{S12}$$

However, in fact, the destination attraction $D_j = \sum_i T_{ij}$ is dependent on the fluxes T_{ij}, resulting in the essential difficulty in solving the Nash equilibrium of DCG . Therefore, we use the iterative algorithm MSA (see **Material and Methods** in the main text) to numerically solve the DCG model. In the MSA iteration, the function to calculate the iterative fluxes $F_{ij}^{(\mathbf{n})}$ is just the Eq. (S12).

If the destination attraction D_j is fixed, the DCG model's potential function is $\phi(S) = \sum_i \sum_j \int_0^{T_{ij}} (\alpha \ln A_j - \beta \ln d_{ij} - \gamma \ln D_j - \ln x)\mathrm{d}x$. For the optimization problem in which $\max \phi(S)$ is subjected to $\sum_j T_{ij} = O_i$ and $\sum_i T_{ij} = D_j$, the Lagrangian expression is

$$\begin{aligned}\max L(T_{ij}) =& \sum_i \sum_j \int_0^{T_{ij}} (\alpha \ln A_j - \beta \ln d_{ij} - \gamma \ln D_j - \ln x)\mathrm{d}x + \\ & \sum_i \lambda_i (\sum_j T_{ij} - O_i) + \sum_j \mu_j (\sum_i T_{ij} - D_j) \\ =& \sum_j \int_0^{D_j} (\alpha \ln A_j - \gamma \ln D_j)\mathrm{d}x - \sum_i \sum_j \int_0^{T_{ij}} (\beta \ln d_{ij} + \ln x)\mathrm{d}x + \\ & \sum_i \lambda_i (\sum_j T_{ij} - O_i) + \sum_j \mu_j (\sum_i T_{ij} - D_j),\end{aligned} \tag{S13}$$

where λ_i and μ_j are Lagrange multipliers. The partial derivative of the Lagrangian expression with respect to T_{ij} is

$$\frac{\partial L}{\partial T_{ij}} = -\beta \ln d_{ij} - \ln T_{ij} + \lambda_i + \mu_j = 0, \tag{S14}$$

therefore

$$T_{ij} = \mathrm{e}^{\lambda_i + \mu_j} d_{ij}^{-\beta}. \tag{S15}$$

Since

$$O_i = \sum_j T_{ij} = \sum_j \mathrm{e}^{\lambda_i + \mu_j} d_{ij}^{-\beta} \tag{S16}$$

and

$$D_j = \sum_i T_{ij} = \sum_i \mathrm{e}^{\lambda_i + \mu_j} d_{ij}^{-\beta}, \tag{S17}$$

we can get

$$\mathrm{e}^{\lambda_i} = O_i / \sum_j \mathrm{e}^{\mu_j} d_{ij}^{-\beta} \tag{S18}$$

and

$$\mathrm{e}^{\mu_j} = D_j / \sum_i \mathrm{e}^{\lambda_i} d_{ij}^{-\beta}. \tag{S19}$$

Let $a_i = \mathrm{e}^{\lambda_i}/O_i$ and $b_j = \mathrm{e}^{\mu_j}/D_j$, Eq. (S15) can be rewritten as

$$T_{ij} = a_i O_i b_j D_j d_{ij}^{-\beta}, \tag{S20}$$

which is the standard doubly-constrained gravity model [S8]. In the actual calculation, a_i and b_j are two sets of interdependent balancing factors, i.e. $a_i = 1/\sum_j b_j D_j d_{ij}^{-\beta}$ and $b_j = 1/\sum_i a_i O_i d_{ij}^{-\beta}$. This means that the calculation of one set requires the values of the other set: start with all $b_j = 1$, solve for $a_i = 1/\sum_j b_j D_j d_{ij}^{-\beta}$ and then use these values to re-estimate the $b_j = 1/\sum_i a_i O_i d_{ij}^{-\beta}$; repeat until convergence of the two sets is achieved [S8].

S3 Other derivations of the gravity model

S3.1 Maximum entropy approach

The earliest gravity model for spatial interaction was developed by analogy with Newton's law of universal gravitation but lacked a rigorous theoretical base. Wilson proposed a maximum entropy approach to deriving the gravity model by maximizing the entropy of a trip distribution [S9]

$$\begin{aligned} \max \ln \Omega = \ & \ln \frac{T!}{\prod_i \prod_j T_{ij}!} \\ \text{s.t.} \quad & \sum_j T_{ij} = O_i \\ & \sum_i T_{ij} = D_i \\ & \sum_i \sum_j T_{ij} C_{ij} = C, \end{aligned} \tag{S21}$$

where Ω is the number of distinct trip arrangements of individuals, T is the total number of trips, T_{ij} is the number of trips from location i to location j, O_i is the total number of departures from i, D_j is the total number of arrivals at j, C_{ij} is the travelling cost from i to j and C is the total travelling cost.

According to the maximum entropy principle, the most likely trip distribution is the distribution with the largest number of microscopic states. Using the Lagrange multiplier method to solve Eq. (S21), we can get

$$T_{ij} = a_i O_i b_j D_j \mathrm{e}^{-\lambda C_{ij}}, \tag{S22}$$

where $a_i = 1/\sum_j b_j D_j \mathrm{e}^{-\lambda C_{ij}}$ and $b_j = 1/\sum_i a_i O_i \mathrm{e}^{-\lambda C_{ij}}$ are interdependent balancing factors. Setting $\lambda C_{ij} = \beta \ln d_{ij}$, where d_{ij} is the distance between i and j, we can get a doubly-constrained gravity model with power distance function

$$T_{ij} = a_i O_i b_j D_j d_{ij}^{-\beta}. \tag{S23}$$

Wilson's maximum entropy derivation offers a theoretical base for the gravity model. However, the maximum entropy principle in statistical physics can only give the most likely macrostate (i.e., the most likely trip distribution matrix $\mathbf{T}$) but cannot describe the individuals' decision processes (i.e., the microscopic mechanism) in the system [S10]. Meanwhile, the total cost in the maximum entropy method is not causally bounded by the theory itself, but determined externally [S11]. As the so-called total cost cannot be estimated in real world, the maximum entropy theory is less practical.

S3.2 Deterministic utility theory

Some scientists described the micro decision-making process of individual spatial interaction (destination choice) using the principle of utility maximization in economics [S10]. Earlier studies used deterministic utility theory to derive the gravity model. The derivation is given in terms of trips made by individuals from a single origin to many destinations [S12]. For an individual k at origin i, assume that there are αm_j persons or things at each destination j with which the individual at i would like to interact per trip, where m_j is the population at j and α is a parameter. Then, k's utility of tripmaking from i to all destinations is

$$U_i^{(k)} = \sum_j U_{ij}^{(k)} = \alpha \sum_j m_j f(T_{ij}^{(k)}), \tag{S24}$$

where $U_i^{(k)}$ is the total utility of individual k at location i of interactions with persons and things at all destinations per unit time, $U_{ij}^{(k)}$ is utility of interactions between individual k at location i and persons or things at destination j per unit time, $T_{ij}^{(k)}$ is the number of trips taken by individual k from i to j per unit time, and f is a function.

An individual's number of trips is constrained by the total cost that the individual can pay,

$$r \sum_j d_{ij}\, T_{ij}^{(k)} \leq \, C_i^{(k)}, \tag{S25}$$

where r is the cost per unit distance travelled and $C_i^{(k)}$ is the total amount of money individual k located at i is willing to spend on travels per unit time.

Setting $f(T_{ij}^{(k)}) = \ln T_{ij}^{(k)}$ and using the Lagrange multiplier method to maximize Eq. (S24) under constraint Eq. (S25), we can derive

$$T_{ij}^{(k)} = \frac{C_i^{(k)}}{r} \cdot \frac{m_j}{\sum\limits_j m_j} \cdot \frac{1}{d_{ij}}. \tag{S26}$$

The total number of trips taken by all individuals from i to j is obtained by summing

the trips from i to j taken by all individuals at i:

$$T_{ij} = \sum_k T_{ij}^{(k)} = \frac{C_i}{r} \cdot \frac{m_j}{\sum\limits_j m_j} \cdot \frac{1}{d_{ij}}, \tag{S27}$$

where C_i is the total amount of money that all individuals at origin i are willing to spend on travels per unit time.

The main problem of this deterministic approach is that the total budget needs to be determined in advance. This is similar to the problem of Wilson's maximum entropy approach, which requires the prior constraint of the total cost. In addition, this method describes the individual's destination selection process over a continuous time period (i.e., the unit time). If the period is short enough and individuals can only complete one trip, then the individuals at a given origin will all select the same destination with the maximum utility, and there will be no dispersion of trips [S10].

S3.3 Random utility theory

Domencich and McFadden applied the random utility theory to many transport-related discrete choice problems [S13], including trip destination choice. In this method, the random utility U_{ij} of a destination j for the individuals starting from origin i is defined as

$$U_{ij} = V_{ij} + \varepsilon_{ij}, \tag{S28}$$

where V_{ij} is a nonstochastic element reflecting the observed attributes of i and j, and ε_{ij} is a random variable describing an unobserved element containing attributes of the alternatives and characteristics of the individual that we are unable to measure.

The individual will choose the destination j that maximized his utility, say

$$U_{ij} > U_{ik}, \forall k \in J - \{j\}, \tag{S29}$$

where J is the set of all candidate destinations.

Since these utility values are stochastic, the choice probability of destination j for any individual at i is given by

$$P_{ij} = \mathrm{Prob}(U_{ij} > U_{ik}, \forall k \in J - \{j\}) = \mathrm{Prob}(V_{ij} + \varepsilon_{ij} > V_{ik} + \varepsilon_{ik}, \forall k \in J - \{j\}). \tag{S30}$$

If the random variables ε_{ij} are independently and identically distributed Gumbel random variables, i.e.,

$$F(\varepsilon_{ij}) = \mathrm{e}^{-\mathrm{e}^{-\varepsilon_{ij}}}, \tag{S31}$$

then, from Eq. (S30), we can get [S14]

$$\begin{aligned}P_{ij} &= \text{Prob}(\varepsilon_{ik} < V_{ij} - V_{ik} + \varepsilon_{ij}, \forall k \in K - \{j\}) \\ &= \text{Prob}(\varepsilon_{ij} = x) \cdot \prod_{k \neq j} \text{Prob}(\varepsilon_{ik} < V_{ij} - V_{ik} + x) \\ &= \int_{-\infty}^{\infty} \frac{\mathrm{d}F(x)}{\mathrm{d}x} F(V_{ij} - V_{ik} + x) \mathrm{d}x \\ &= \int_{-\infty}^{\infty} \mathrm{e}^{-x} \mathrm{e}^{-\mathrm{e}^{-x}} \prod_{k \neq j} \mathrm{e}^{-\mathrm{e}^{-(V_{ij} - V_{ik} + x)}} \mathrm{d}x.\end{aligned} \tag{S32}$$

Noting that $V_{ij} - V_{ij} = 0$, so Eq. (S32) can be written as

$$\begin{aligned}P_{ij} &= \int_{-\infty}^{\infty} \mathrm{e}^{-x} \prod_{k} \mathrm{e}^{-\mathrm{e}^{-(V_{ij} - V_{ik} + x)}} \mathrm{d}x \\ &= \int_{-\infty}^{\infty} \mathrm{e}^{-\sum_k \mathrm{e}^{-(V_{ij} - V_{ik} + x)}} \mathrm{e}^{-x} \mathrm{d}x \\ &= \int_{-\infty}^{\infty} \mathrm{e}^{-\mathrm{e}^{-x} \sum_k \mathrm{e}^{-(V_{ij} - V_{ik})}} \mathrm{e}^{-x} \mathrm{d}x.\end{aligned} \tag{S33}$$

Define $t = \mathrm{e}^{-x}$ such that $\mathrm{d}t = -\mathrm{e}^{-x}\mathrm{d}x$. When $x = \infty$, $t = \mathrm{e}^{-\infty} = 0$ and when $x = -\infty$, $y = \mathrm{e}^{\infty} = \infty$. Therefore, Eq. (S33) can be written as

$$\begin{aligned}P_{ij} &= \int_{\infty}^{0} \mathrm{e}^{-t \sum_k \mathrm{e}^{-(V_{ij} - V_{ik})}} \cdot (-\mathrm{d}t) \\ &= \int_{0}^{\infty} \mathrm{e}^{-t \sum_k \mathrm{e}^{-(V_{ij} - V_{ik})}} \mathrm{d}t \\ &= \left. \frac{\mathrm{e}^{-t \sum_k \mathrm{e}^{-(V_{ij} - V_{ik})}}}{-\sum_k \mathrm{e}^{-(V_{ij} - V_{ik})}} \right|_0^{\infty} \\ &= \frac{\mathrm{e}^{-\infty} - \mathrm{e}^0}{-\sum_k \mathrm{e}^{-(V_{ij} - V_{ik})}} \\ &= \frac{\mathrm{e}^{V_{ij}}}{\sum_k \mathrm{e}^{V_{ik}}},\end{aligned} \tag{S34}$$

which is the Logit model usually used in transport modal choice [S8]. If we set $V_{ij} = \ln m_j - \beta \ln d_{ij}$, we can get an origin-constrained gravity model

$$T_{ij} = O_i P_{ij} = O_i \frac{\mathrm{e}^{V_{ij}}}{\sum\limits_j \mathrm{e}^{V_{ij}}} = O_i \frac{m_j d_{ij}^{-\beta}}{\sum\limits_j m_j d_{ij}^{-\beta}}. \tag{S35}$$

Random utility theory accounts for the dispersion of trips from an origin and does not require a predetermined total budget. Therefore, the gravity model based on random utility theory seems superior to other approaches based on deterministic utility theory or maximum entropy theory [S10]. However, random utility theory asks for an oversubtle condition, namely the existence of an unobserved variable ε_{ij} that has to obey the independent and identical Gumbel distribution.

References

[S1] Cho, E., Myers, S. A. & Leskovec, J. Friendship and mobility: User movement in location-based social networks. *Proceedings of the 17th ACM SIGKDD International Conference on Knowledge Discovery and Data Mining,* 1082-1090 (2011).

[S2] Bao, J., Zheng, Y. & Mokbel, M. F. Location-based and preference-aware recommendation using sparse geo-social networking data. *ACM the 20th International Conference on Advances in Geographic Information Systems,* 199-208 (2012).

[S3] Darling, D. A. The Kolmogorov-Smirnov, Cramer-von Mises tests *Ann. Math. Stat.* **28,** 823-837 (1957).

[S4] Rosenthal, R. W. A class of games possessing pure-strategy Nash equilibria. *Int. J. Game Theory* **2,** 65-67 (1973).

[S5] Monderer, D. & Shapley, L. S. Potential games. *Games Econ. Behav.* **14,** 124-143 (1996).

[S6] Voorneveld, M., Borm, P., Van Megen, F., Tijs, S. & Facchini, G. Congestion games and potentials reconsidered. *Int. Game Theory Rev.* **1,** 283-299 (1999).

[S7] Vöcking, B. & Aachen, R. Congestion games and potentials reconsidered. *Proceedings of the 2nd Algorithms and Complexity in Durham Workshop,* 9-20 (2006).

[S8] Ortúzar, J. D. & Willumsen, L. G. *Modelling transport* (John Wiley & Sons, New York, 2011).

[S9] Wilson, A. G. A statistical theory of spatial distribution models. *Transport. Res.* **1,** 253-269 (1967).

[S10] Sheppard, E. S. Theoretical underpinnings of the gravity hypothesis. *Geogr. Anal.* **10(4),** 386-402 (1978).

[S11] Hua, C. I. & Porell, F. A critical review of the development of the gravity model. *Int. Regional Sci. Rev.* **4(2),** 97-126 (1979).

[S12] Niedercorn, J. H., Bechdolt & Jr B. V. An economic derivation of the "gravity law" of spatial interaction. *J. Regional Sci.* **9(2),** 273-282 (1969).

[S13] Domencich, T. A. & Mcfadden, D. *Urban travel demand: A behavioral analysis* (North-Holland, Amsterdam, 1975).

[S14] Train, K. E. *Discrete choice methods with simulation* (Cambridge University Press, Cambridge, 2009).

Table S1: **Estimated parameters in parameterised spatial interaction models.** α-DCG, β-DCG and γ-DCG are the parameters in DCG model. α-G2 and β-G2 are the parameters in Gravity 2 model. β-G1 is the parameter in Gravity 1 model and α-IO is the parameter in IO model.

Data set	α-DCG	β-DCG	γ-DCG	α-G2	β-G2	β-G1	α-IO
Abidjan	2.99	2.53	2.28	0.88	2.49	2.43	3.04×10^{-5}
China	3.85	1.07	2.68	1.10	0.91	0.96	8.52×10^{-7}
US (migration)	4.45	0.60	2.88	1.19	0.56	0.59	7.73×10^{-7}
Japan	3.00	0.72	2.21	0.95	0.54	0.52	1.59×10^{-4}
UK	1.97	1.89	0.99	1.03	1.86	1.88	1.72×10^{-4}
Belgium	2.80	1.28	2.01	1.01	1.17	1.20	1.31×10^{-4}
New York	2.99	0.70	2.05	0.92	0.64	0.54	2.13×10^{-5}
Los Angeles	2.85	1.13	2.09	0.85	1.08	1.05	5.60×10^{-5}
Oslo	1.86	1.03	1.01	0.90	0.87	0.76	5.87×10^{-5}

Table S2: **Two-sample KS statistics of the travel distance distribution predicted by model and that observed from real data.** The bold number is the minimum value of KS statistic in a line.

Data set	DCG	Gravity 1	Gravity 2	IO	PWO	Radiation
Abidjan	0.042	**0.032**	0.044	0.161	0.135	0.296
China	**0.077**	0.077	0.099	0.151	0.102	0.286
US (migration)	0.031	**0.023**	0.031	0.052	0.090	0.445
Japan	0.056	0.087	0.084	**0.032**	0.068	0.284
UK	**0.029**	0.048	0.044	0.056	0.061	0.248
Belgium	**0.064**	0.074	0.071	0.074	0.108	0.282
New York	**0.006**	0.006	0.007	0.012	0.023	0.101
Los Angeles	0.005	**0.003**	0.004	0.007	0.020	0.069
Oslo	0.022	0.014	**0.009**	0.026	0.102	0.500

Table S3: **Two-sample KS statistics of the destination attraction distribution predicted by model and that observed from real data.** The bold number is the minimum value of KS statistic in a line.

Data set	DCG	Gravity 1	Gravity 2	IO	PWO	Radiation
Abidjan	**0.032**	0.096	0.068	0.155	0.087	0.064
China	0.103	**0.082**	0.171	0.103	0.147	0.206
US (migration)	0.137	**0.098**	0.157	0.118	0.137	0.216
Japan	**0.064**	0.191	0.149	0.277	0.277	0.170
UK	**0.085**	0.118	0.129	0.188	0.141	0.102
Belgium	**0.070**	0.116	0.116	0.140	0.209	0.093
New York	0.097	0.200	0.133	0.313	0.246	**0.082**
Los Angeles	**0.024**	0.169	0.093	0.220	0.164	0.036
Oslo	0.037	0.100	0.074	0.140	0.154	**0.026**

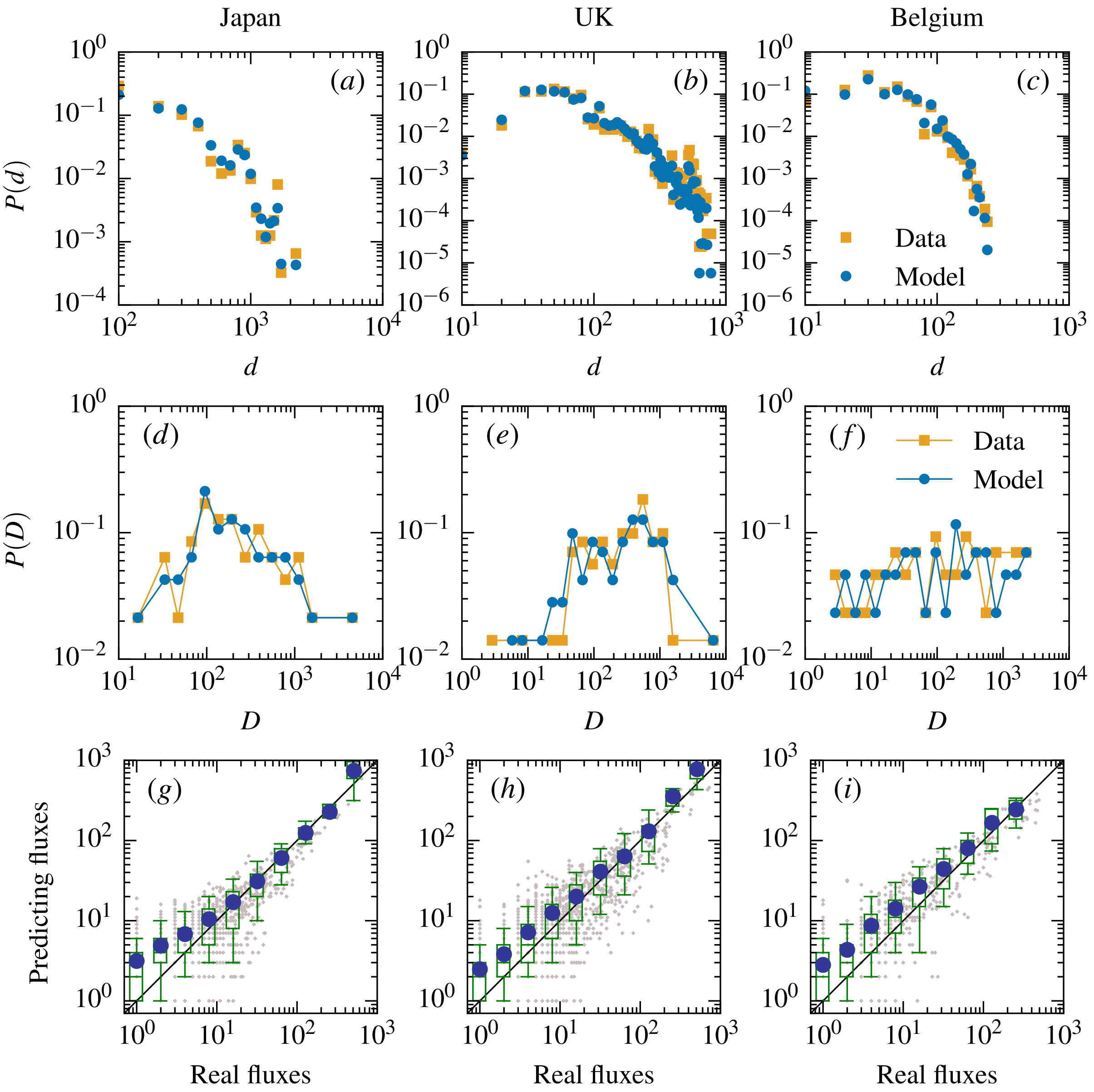

Figure S1: **Comparing the prediction of DCG model and the empirical data of intercity travels**. (*a*-*c*) Predicted and real distributions of travel distances $P(d)$. (*d*-*f*) Predicted and real distributions of locations's attracted travels $P(D)$. (*g*-*i*) Predicted and observed fluxes.

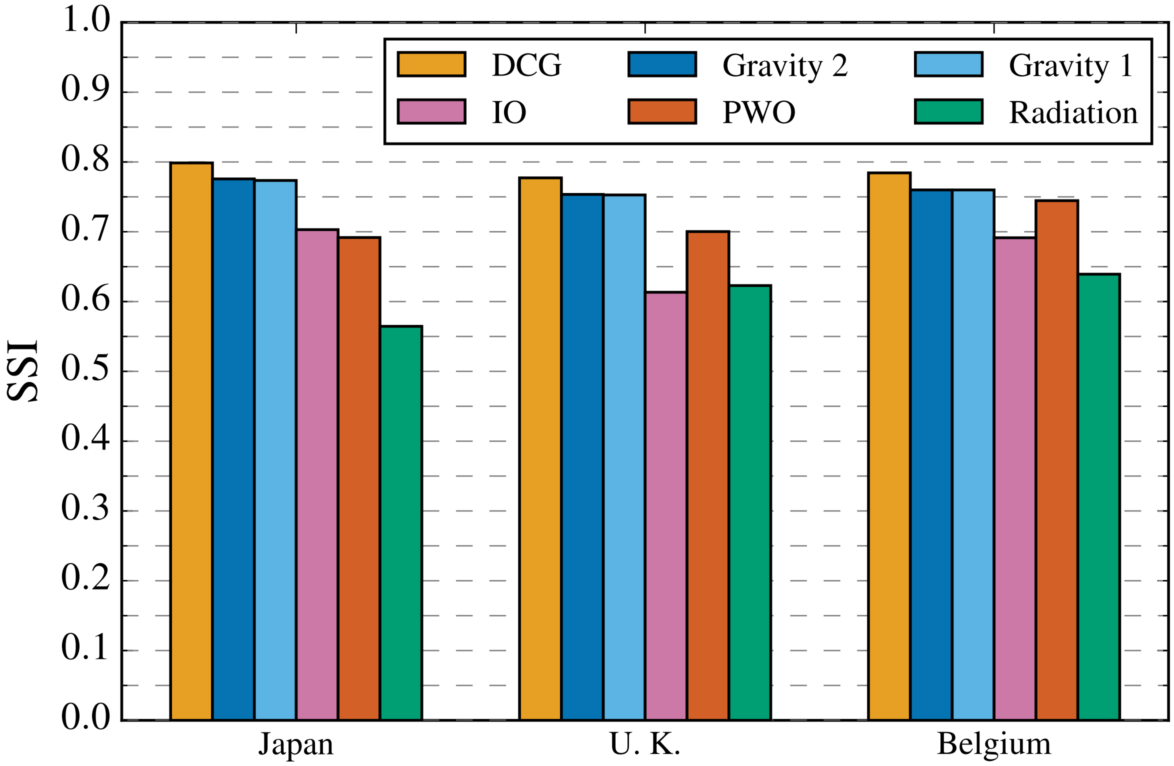

Figure S2: **Comparing predicting accuracy of the DCG model and benchmark models for intercity travels.**

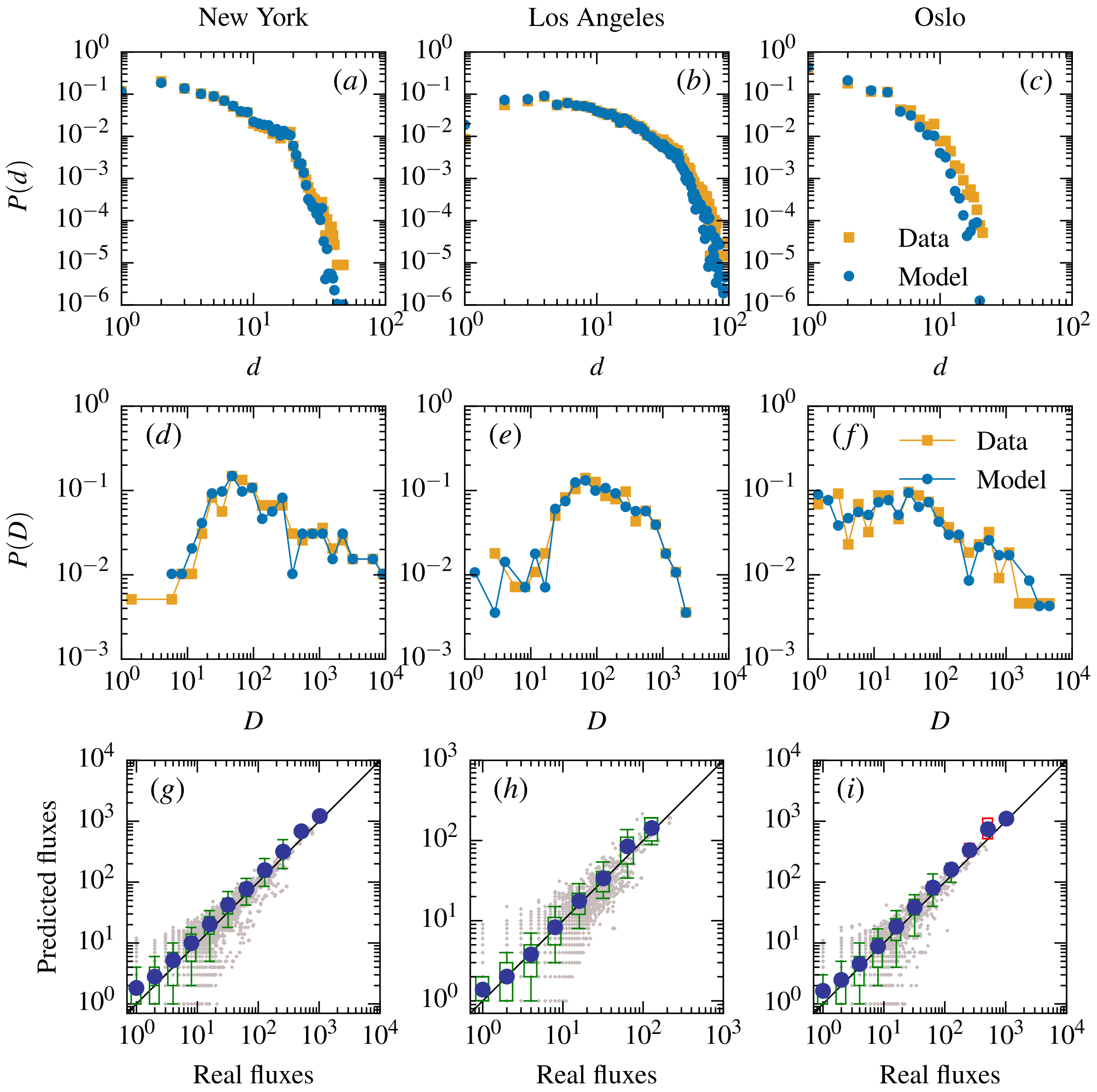

Figure S3: **Comparing the prediction of DCG model and the empirical data of intracity trips**. (*a*-*c*) Predicted and real distributions of travel distances $P(d)$. (*d*-*f*) Predicted and real distributions of locations's attracted travels $P(D)$. (*g*-*i*) Predicted and observed fluxes.

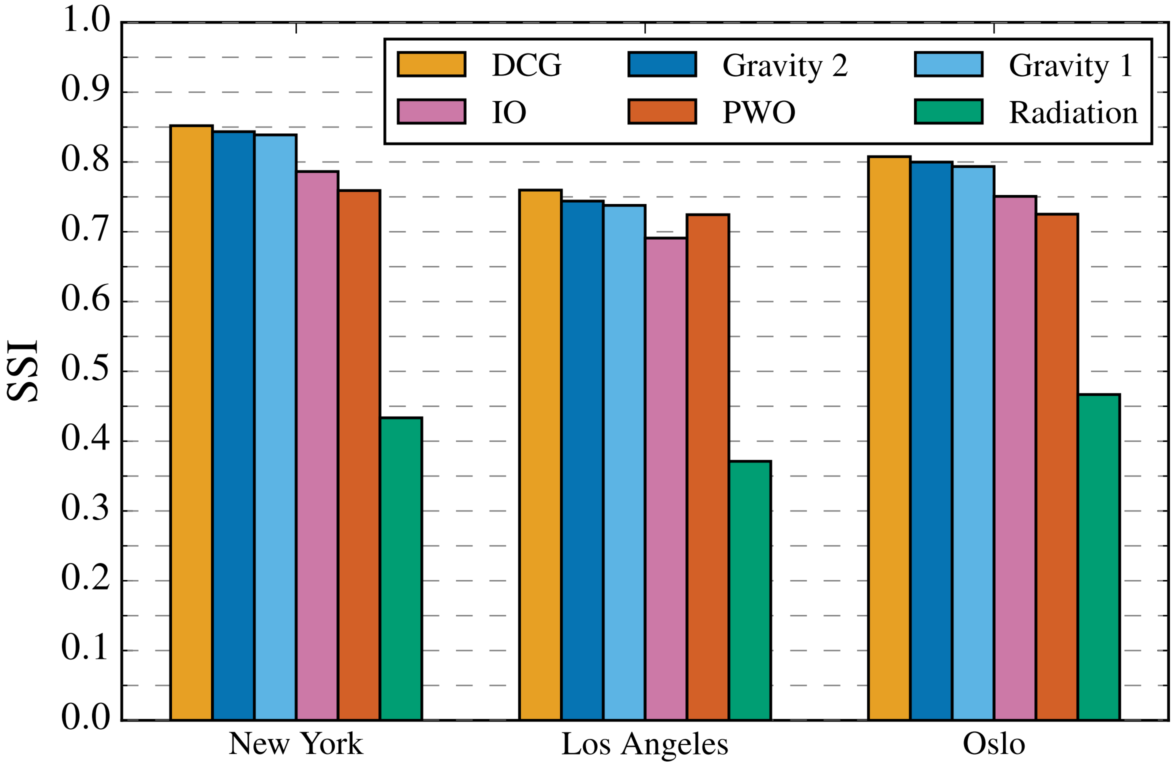

Figure S4: **Comparing predicting accuracy of the DCG model and benchmark models for intracity trips.**

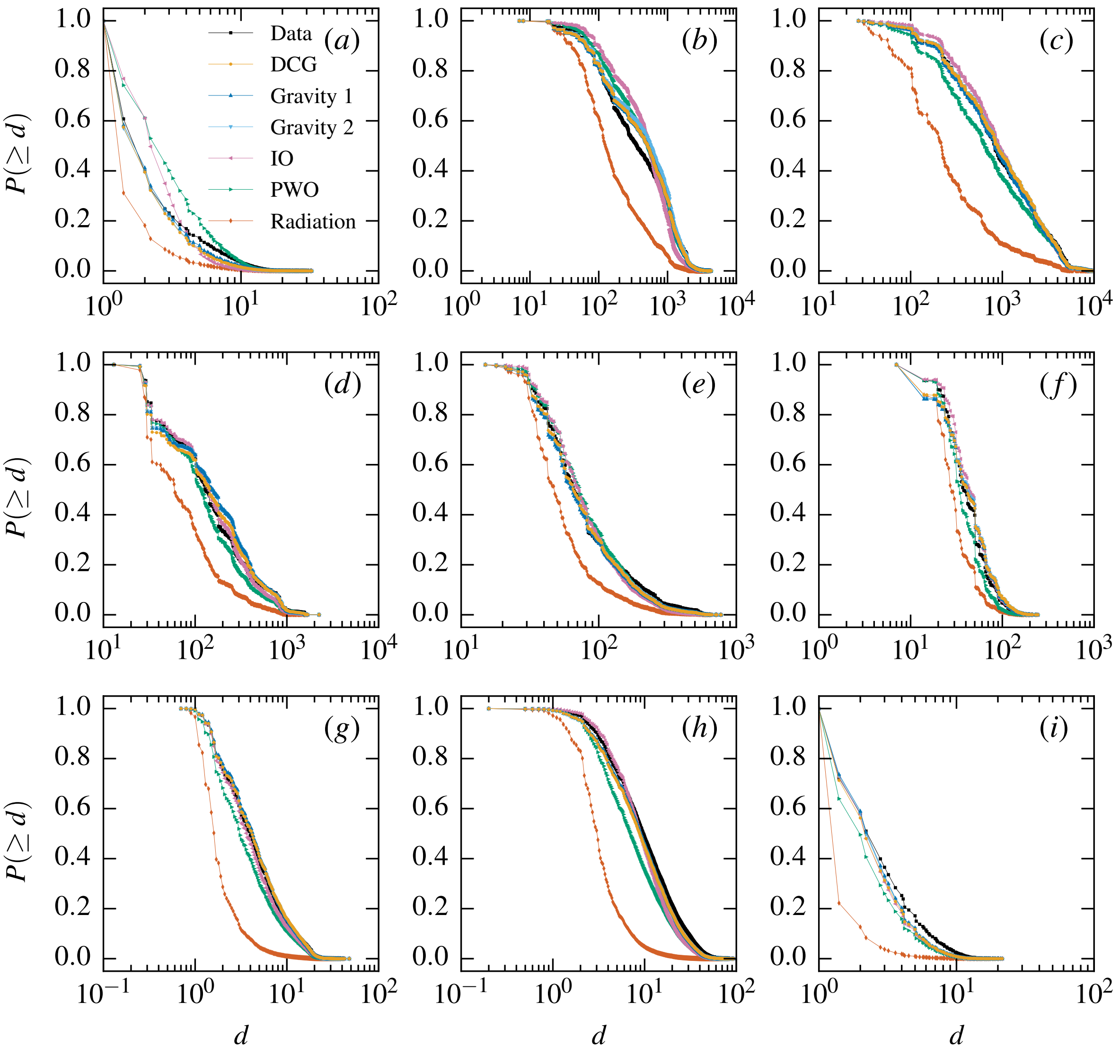

Figure S5: **Comparing predicting accuracy of the DCG model and benchmark models in terms of travel distance distribution.** (a) Abidjan. (b) China. (c) US. (d) Japan. (e) UK. (f) Belgium. (g) New York. (h) Los Angeles. (i) Oslo.

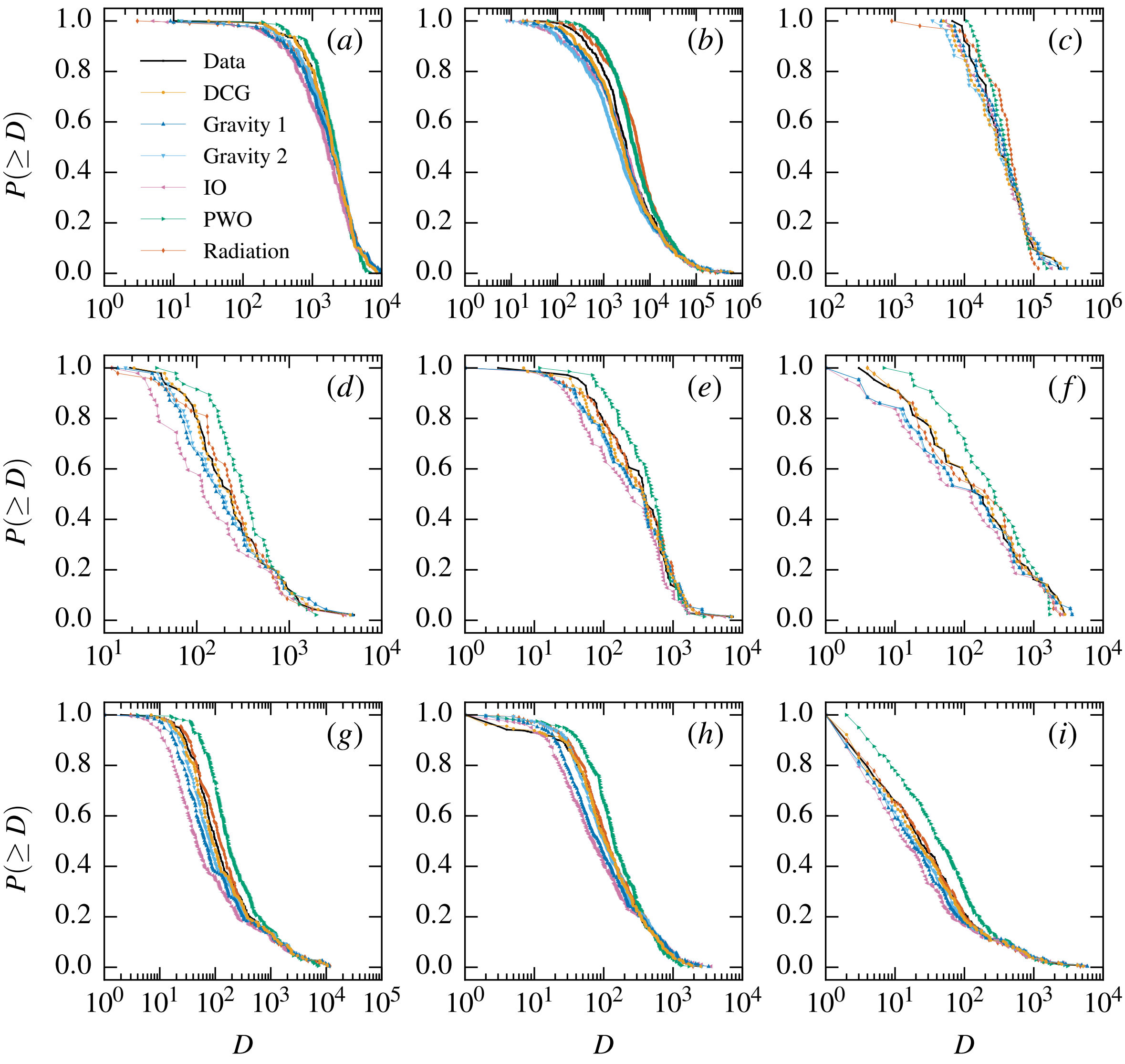

Figure S6: **Comparing predicting accuracy of the DCG model and benchmark models in terms of destination attraction distribution.** (*a*) Abidjan. (*b*) China. (*c*) US. (*d*) Japan. (*e*) UK. (*f*) Belgium. (*g*) New York. (*h*) Los Angeles. (*i*) Oslo.
